# Calculation of Transpulmonary Pressure From Regional Ventilation Displayed by Electrical Impedance Tomography in Acute Respiratory Distress Syndrome

**DOI:** 10.3389/fphys.2021.693736

**Published:** 2021-07-19

**Authors:** Gaetano Scaramuzzo, Savino Spadaro, Elena Spinelli, Andreas D. Waldmann, Stephan H. Bohm, Irene Ottaviani, Federica Montanaro, Lorenzo Gamberini, Elisabetta Marangoni, Tommaso Mauri, Carlo Alberto Volta

**Affiliations:** ^1^Department of Translational Medicine and for Romagna, University of Ferrara, Ferrara, Italy; ^2^Department of Anesthesia, Critical Care and Emergency, Fondazione IRCCS Ca' Granda Ospedale Maggiore Policlinico, Milan, Italy; ^3^Department of Anesthesiology and Intensive Care Medicine, Rostock University Medical Center, Rostock, Germany; ^4^Department of Anaesthesia, Intensive Care and Prehospital Emergency, Ospedale Maggiore Carlo Alberto Pizzardi, Bologna, Italy; ^5^Department of Pathophysiology and Transplant, University of Milan, Milan, Italy

**Keywords:** driving pressure, transpulmonary pressure, acute respiratory distress syndrome, precision medicine, electric impedance tomography

## Abstract

Transpulmonary driving pressure (DP_L_) corresponds to the cyclical stress imposed on the lung parenchyma during tidal breathing and, therefore, can be used to assess the risk of ventilator-induced lung injury (VILI). Its measurement at the bedside requires the use of esophageal pressure (Peso), which is sometimes technically challenging. Recently, it has been demonstrated how in an animal model of ARDS, the transpulmonary pressure (P_L_) measured with Peso calculated with the absolute values method (P_L_ = Paw—Peso) is equivalent to the transpulmonary pressure directly measured using pleural sensors in the central-dependent part of the lung. We hypothesized that, since the P_L_ derived from Peso reflects the regional behavior of the lung, it could exist a relationship between regional parameters measured by electrical impedance tomography (EIT) and driving P_L_ (DP_L_). Moreover, we explored if, by integrating airways pressure data and EIT data, it could be possible to estimate non-invasively DP_L_ and consequently lung elastance (EL) and elastance-derived inspiratory P_L_ (PI). We analyzed 59 measurements from 20 patients with ARDS. There was a significant intra-patient correlation between EIT derived regional compliance in regions of interest (ROI1) (*r* = 0.5, *p* = 0.001), ROI2 (*r* = −0.68, *p* < 0.001), and ROI3 (*r* = −0.4, *p* = 0.002), and DP_L_. A multiple linear regression successfully predicted DP_L_ based on respiratory system elastance (Ers), ideal body weight (IBW), roi1%, roi2%, and roi3% (*R*^2^ = 0.84, *p* < 0.001). The corresponding Bland-Altmann analysis showed a bias of −1.4e-007 cmH_2_O and limits of agreement (LoA) of −2.4–2.4 cmH_2_O. EL and PI calculated using EIT showed good agreement (*R*^2^ = 0.89, *p* < 0.001 and *R*^2^ = 0.75, *p* < 0.001) with the esophageal derived correspondent variables. In conclusion, DP_L_ has a good correlation with EIT-derived parameters in the central lung. DP_L_, PI, and EL can be estimated with good accuracy non-invasively combining information coming from EIT and airway pressure.

## Introduction

Monitoring transpulmonary pressure can be important in patients affected by acute respiratory distress syndrome (ARDS) (ARDS Definition Task Force et al., 2012; Chiumello et al., 2014). Pressure measured at airway opening, indeed, does not yield information about the different components of the respiratory system, i.e., the chest wall and the lung. Chest wall and lung elastance (EL) can differ unpredictably such that, patients having the same airway pressures can have significantly different transpulmonary pressures (Gattinoni et al., 2004; Chiumello et al., 2008). The current approach to evaluate transpulmonary pressure (P_L_) is based on the use of esophageal pressure (Peso) and assumes that it could be a good surrogate for pleural pressure (Grieco et al., 2017). The transpulmonary driving pressure (DP_L_), i.e., the variation of transpulmonary pressure between end-expiration and end-inspiration, corresponds to the cyclical stress imposed on the lung parenchyma. Unlike driving pressure calculated from airway opening pressure (DP), DP_L_ is the pressure imposed on the lung during the tidal breathing, since it does not consider the amount of pressure needed to overcome the chest-wall compartment (Loring and Malhotra, 2015). Evaluating DP_L_ can be important to limit the stress on the lung parenchyma and, therefore, may be useful to monitor the risk for ventilator-induced lung injury (VILI). Recently, it has been demonstrated in an animal model of ARDS how transpulmonary pressure calculated with the classical absolute subtractive method (i.e., P_L_ = Paw—Peso) corresponds to the transpulmonary pressure in the central to dependent lung (Yoshida et al., 2018). When we consider transpulmonary pressure, we should consider that its value is not unique along with the whole lung but changes regionally according to regional differences in pleural pressures which reflects the forces acting in favor of lung collapse or opening (regional heterogeneity of core disease, gravitational distribution of edema, and mediastinum weight) (Silva and Gama de Abreu, 2018). Electrical impedance tomography (EIT) is a non-invasive monitoring technique that can help to monitor regional lung ventilation distribution at the bedside (Frerichs et al., 2017; Yoshida et al., 2019; Scaramuzzo et al., 2020c). Since the P_L_ derived from Peso reflects the behavior of the central to dependent part of the lung, we aimed to evaluate the relationship between regional mechanics variables, derived by EIT, and transpulmonary pressure in patients affected by ARDS. Moreover, we tested if, by integrating the information from EIT and airway opening pressure, we could predict non-invasively DP_L_. Finally, we wanted to verify if EL and the elastance-based inspiratory P_L_ derived by EIT, agree with the one classically calculated using esophageal manometry.

## Methods

This is a secondary analysis of data collected from a database of patients affected by ARDS enrolled in a previous study (Scaramuzzo et al., 2020b) in two university hospital intensive care units (Arcispedale Sant'Anna Hospital, Ferrara, Italy and at Cà Granda IRCCS, Milano). The study was approved by the ethics committee of the Sant' Anna Hospital, Ferrara, Italy (Protocol n. 171098) and Milan (protocol no. 625_2018). The selection criteria for the current data analysis were: ARDS according to the Berlin criteria (ARDS Definition Task Force et al., 2012), EIT images for at least 2 min containing an end-inspiratory and end-expiratory pauses, simultaneous recording of airway opening pressure, and airway flow and Peso. Enrolled patients were all mechanically ventilated in volume-controlled ventilation (VCV) with a tidal volume (TV) = 6 ml/kg/IBW. An occlusion test was performed for each patient (Baydur et al., 1982) to assess the correct positioning of the esophageal balloon. All patients were sedated and paralyzed, as per clinical decision and no recruitment maneuver was performed before the measurements. We collected three measures from each patient at three different levels of PEEP, based on clinical practice, transpulmonary pressure, and EIT. The method for PEEP setting guided by EIT and P_L_ was described previously by Scaramuzzo et al. (2020b).

### Respiratory Mechanics

The following mechanical measurements were collected from the airway opening pressure end-inspiratory and end-expiratory pauses: TV, total positive end-expiratory pressure (PEEP), peak pressure (peak), and plateau pressure (Pplat). The DP of the respiratory system was calculated as Pplat-PEEP. The elastance of the respiratory system (Ers) was calculated as *Ers* = DP/TV and was expressed in cmH_2_O/L. Transpulmonary pressure (P_L_) was calculated as the difference between airway pressure and Peso (P_L_ = Pao–Peso) and DP_L_ as the difference between end-inspiratory and end-expiratory P_L_. EL was calculated as *EL* = DP_L_/TV while chest-wall elastance (Ecw) as *Ecw* = Ers–EL. The elastance-derived inspiratory P_L_ (PI) was calculated as *PI* = Pplat–[Pplat^*^(Ecw/Ers)].

### EIT Analysis

An average of 10 respiratory acts was used to analyze EIT and the regional analysis was conducted by dividing the EIT image into four craniocaudal regions of interest (ROI_N_; ROI1: most ventral; ROI4: most dorsal). The percentage of tidal ventilation (ROI%_N_) in four ROIs was calculated as the fraction of tidal delivered to the ROI in the analyzed acts and was expressed in percentage (Frerichs et al., 2017). The weighted regional compliance in the four ROIs was calculated as follows:

$$RC_{ROI}=\frac{\frac{TV\times ROI\%}{DP}}{IBW}\;$$

and expressed as ml/cmH_2_O/kg of ideal body weight (IBW).

### Statistical Analysis

Data are expressed as median [IQR]. Repeated measures correlation (rmcorr) [Bakdash and Marusich (2020)]. R package version 0.4.1. https://CRAN.R-project.org/package=rmcorr) was used to test correlation among variables with repeated measures. To predict measured DP_L_, a linear regression analysis [panel linear model, plm (Croissant and Millo, 2008)] accounting for the longitudinal characteristic of the data (cross-sectional time-series data) was used. Time of sampling and patient ID were considered as fixed factors. The pooled OLS estimation method was used, and the following variables were entered as predictors, based on their clinical meaningfulness and the results of rmcorr: IBW, Ers (derived by TV and DP), roi1%, roi2, and roi3%. The resulting EIT-derived driving transpulmonary pressure (named DP_L, EIT_) was used to calculate EL as EL_EIT_= TV/DP_L, EIT_. The EIT PI was calculated as PI_EIT_ = Pplat—[Pplat((Ers–EL_EIT_) /Ers)]. Bias and limits of agreement (LOA) with mean bias ± 2 sds were calculated as per the Bland-Altman approach (Bland and Altman, 1986) between EIT- and Peso-derived DP_L_, EL, and PI. To evaluate if the PEEP titration technique or the number of quadrants infiltrated at the chest x-ray could affect the agreement between the two techniques, we performed an additional linear regression between EIT and Peso-derived DP_L_ (as shown in Supplementary Material). The statistical analysis was conducted using GraphPad Prism 8.4.3 for Windows (GraphPad Software, San Diego, California USA, www.graphpad.com) and R 4.0.4 (R Foundation for Statistical Computing, Vienna, Austria) [R Core Team (2021). R: A language and environment for statistical computing. R Foundation for Statistical Computing, Vienna, Austria. URL https://www.R-project.org/.]. *P*-values < 0.05 were considered statistically significant.

## Results

### Population Characteristics

Of the 60 measurements considered for the analysis, 59 were analyzed (one excluded after quality check). The measures were derived from 20 patients, 13 males and 7 females, aged 63 [53–72] years with a median body mass index (BMI) of 28 [24–33] kg/m^2^, and a PaO_2_/FiO_2_ of 149 [96–211], and average PEEP 13[9.2–15] cmH_2_O. Each patient had three measures in which PEEP was set according to three different methods (clinical practice, transpulmonary pressure, and EIT). The characteristics of the population are resumed in Table 1. Additional information on lung mechanics and the effect of PEEP titration on lung recruitment/derecruitment has been already published previously by Scaramuzzo et al. (2020b).

**Table 1:** Main characteristics of the patients and the pooled measurements.

**Gender**	***M* = 13; *F* = 7**
Age (years)	63 [53–72]
BMI (Kg/m^2^)^*^	28 [24–33]
SAPS II	53 [45–66]
Days from ICU admission	4 [2–5]
PaO2/FiO2^*^	149 [96–211]
PaCO2 (mmHg)^*^	57 [47–68]
FiO2 (%)^*^	50 [43–60]
Respiratory rate (bpm)	19 [15–24]
Tidal volume (ml/kg IBW)	6.3 [6.1–7.0]
Mild/Moderate/Severe ARDS	6/9/5
Tidal volume (ml)	375 [346–440]
Respiratory rate (acts/min)	19[16–24]
Peak pressure (cmH2O)	31[28–39]
Plateau pressure (cmH2O)	23[19–28]
PEEP (cmH2O)	13 [9.2–15]
Driving pressure (cmH2O)	10 [8.7–13]
End inspiratory P_L_ (cmH2O)	8.7 [5.6–13]
End-expiratory P_L_ (cmH2O)	1.3 [-0.27–3.3]
Transpulmonary driving pressure (cmH2O)	7 [5.8–9.1]
RS Elastance (cmH2O/L)	27 [21–33]
Lung elastance (cmH2O/L)	18 [14–23]
ROI1 tidal distribution (%)	19 [15–22]
ROI2 tidal distribution (%)	37 [33–42]
ROI3 tidal distribution (%)	31 [27–35]
ROI4 tidal distribution (%)	13 [8.3–17]
Regional compliance ROI1 (ml/cmH2O/kg)	0.12 [0.096–0.15]
Regional compliance ROI2 (ml/cmH2O/kg)	0.25 [0.18–0.3]
Regional compliance ROI2 (ml/cmH2O/kg)	0.19 [0.15–0.25]
Regional compliance ROI2 (ml/cmH2O/kg)	0.072 [0.043–0.12]

*BMI, body mass index; SAPSII, simplified acute physiology score II; ICU, intensive care unit; _PaO2_/_FiO2_, partial pressure of arterial oxygen on the inspired fraction of oxygen ratio; PEEP, positive end-expiratory pressure; MAP, mean arterial blood pressure; IBW, ideal body weight; P_L_, transpulmonary pressure; RS, respiratory system*.

* *at ICU admission*.

### Correlation

**Figure 1:**
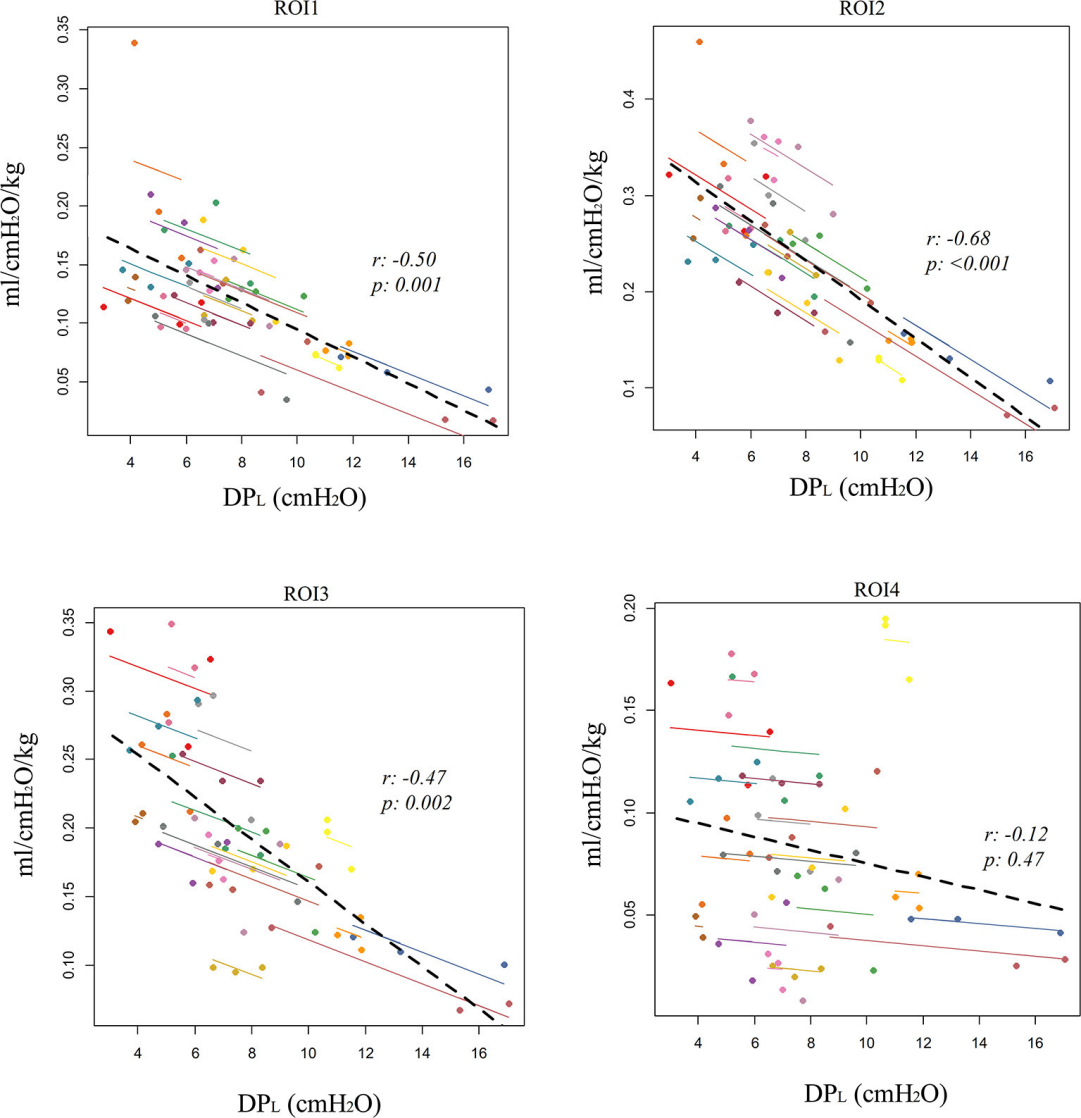
Correlation between electrical impedance tomography (EIT)-derived regional compliance in the four craniocaudal regions of interest (ROI1, ventral lung; ROI4, dorsal lung) and esophageal-derived transpulmonary driving pressure (DP_L_). Repeated measures correlation (rmcorr).

The repeated measures correlation analysis showed a significant intra-patient correlation between DP_L_ and regional tidal distribution, which was negative in ROI1 (*r* = −0.35, *p* = 0.03) and ROI2(*r* = −0.45, *p* = 0.003) and positive in ROI3 (*r* = 0.4, *p* = 0.01) and ROI4 (*r* = 0.4, *p* = 0.01, Table 2). A stronger correlation was found with regional compliance in ROI1 (*r* = −0.5, *p* = 0.001), ROI2 (*r* = −0.68, *p* < 0.001), and ROI3 (*r* = −0.47, *p* = 0.002), while there was no significant correlation between DP_L_ and regional compliance in ROI4 (*r* = −0.12, *p* = 0.47, Table 2; Figure 1).

**Table 2:** Repeated measures correlation (rmcorr) analysis between electrical impedance tomography (EIT)- derived and esophageal-derived parameters.

	**Intra-patient correlation coefficient**	***p*-value**
Relative ventilation, ROI1 (%)	−0.35	0.03
Relative ventilation, ROI2 (%)	−0.45	0.003
Relative ventilation, ROI3 (%)	0.40	0.01
Relative ventilation, ROI4 (%)	0.40	0.01
Regional compliance/IBW (ml/cmH_2_O/kg)—ROI1	−0.50	0.001
Regional compliance/IBW (ml/cmH_2_O/kg)—ROI2	−0.68	<0.001
Regional compliance/IBW (ml/cmH_2_O/kg)—ROI3	−0.47	0.002
Regional compliance/IBW (ml/cmH_2_O/kg)—ROI4	−0.12	0.47

*Correlation coefficients for rmcorr analysis between EIT and esophageal-derived measures*.

### EIT Derived Parameters Calculation

**Figure 2:**
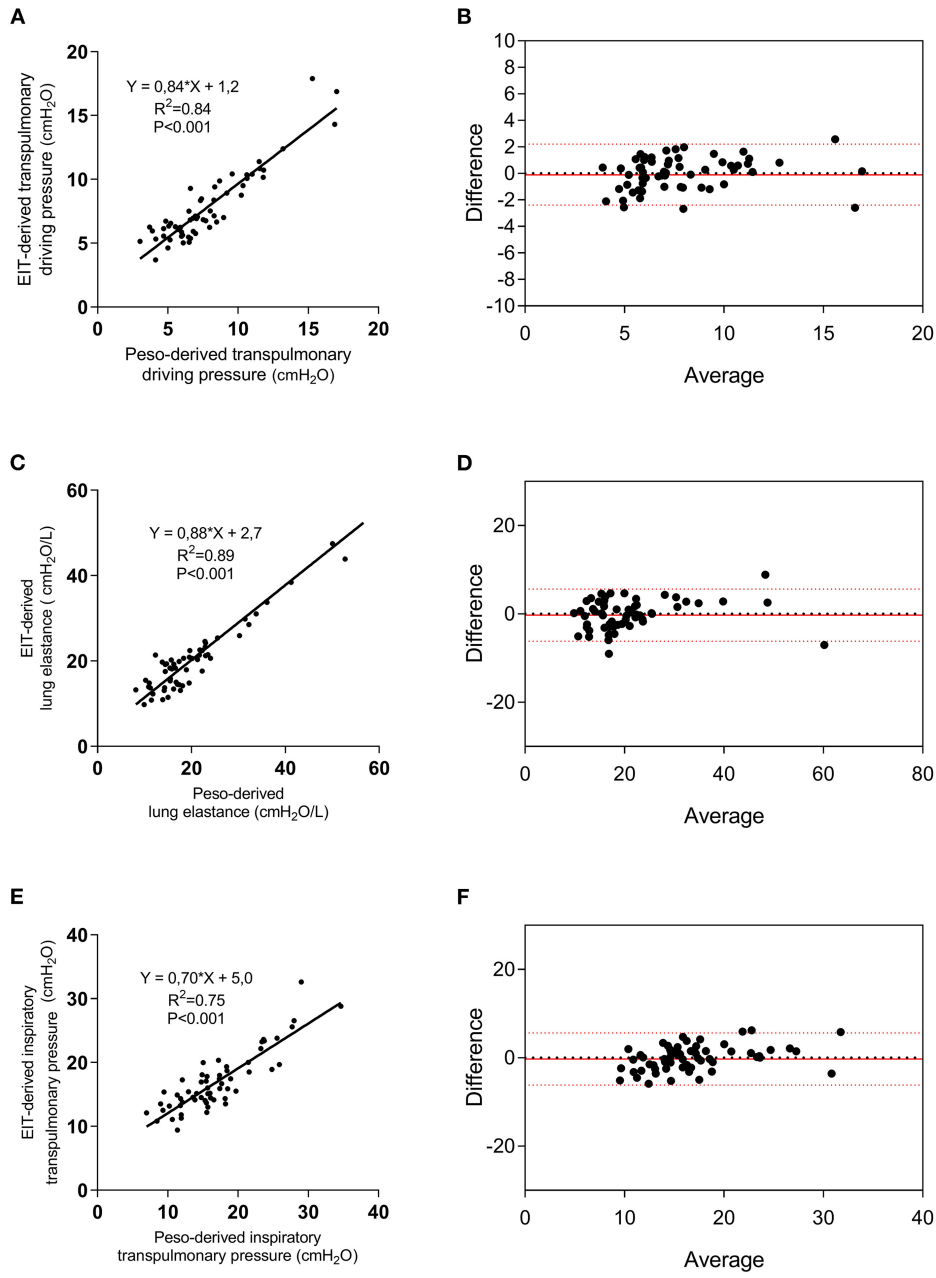
Electrical impedance tomography derived and measured DP_L_, lung elastance (EL), and elastance-derived inspiratory lung pressure. Linear regression between EIT-derived and measured DP_L_ **(A)**, EL **(C)**, and lung inspiratory pressure **(E)**, and relative Bland-Altmann plots **(B,D,F)**. EIT, electrical impedance tomography; Peso, esophageal pressure.

Five regressors were used to perform the linear regression with DP_L_ as the dependent variable: Ers, IBW, roi%1, roi%2, and roi%3. A significant regression was found, with an R^2^ of 0.84 (*p* < 0.001) and predicted DP_L_ (DP_L, EIT_) was equal to DP_L, EIT_ = k + α·IBW + β·Ers + γ·roi1% + δ·roi2% + e· roi3% being *k* = 16.64; α = 0.074683; β = 0.230941; γ = −0.21449; δ = −0.15974; *e* = −0.32996 (Figure 2A; Supplementary Table S1). The corresponding Bland-Altmann between the EIT and Peso-derived measures showed a bias of −1.4e-007 and an LoA of −2.4–2.4 cmH_2_O (Figure 2B). The linear regression between EL_EIT_, and EL resulted in an *R*^2^ = 0.89 (*p* < 0.0001), while the corresponding Bland-Altmann analysis showed a bias of −0.11 ± and an LoA of −6.8–6.5 cmH2O/L (Figures 2C,D). The EIT-derived inspiratory PL predicted well the corresponding Peso derived value (*R*^2^ = 0.75, *p* < 0.0001) and with a good agreement [bias of −0.007 ± and an LoA of −5.6–5.6 cmH_2_O/L (Figures 2E,F)]. The PEEP titration technique did not provide different results in terms of the agreement between the two techniques (Supplementary Figure S2), but in patients with a higher number of quadrants infiltrated at the chest x-ray, the agreement was higher (Supplementary Figure S3).

## Discussion

In this study, we investigated the relationship between the EIT-derived parameters and DP_L_ in patients affected by ARDS. We found that regional ventilation distribution correlates well with DP_L_, especially in the central part of the lung. The highest correlation was found with regional compliance in the ventral-central part of the lung. Moreover, by using EIT and airway opening pressure derived data, we were able to predict DP_L_, EL and lung inspiratory transpulmonary pressure with good accuracy.

Lung monitoring has been increasingly used in the last few years to personalize mechanical ventilation (Pereira et al., 2018; Beitler et al., 2019; Scaramuzzo et al., 2020b) in different settings, especially in ARDS. ARDS requires, indeed, more precise fine-tuned ventilation, since the wide and unpredictable characteristics of the disease, especially the amount of alveolar and interstitial edema, make it difficult to develop a standard that fits all the patients and conditions. Personalizing mechanical ventilation in patients affected by ARDS aims to keep under control different variables, each one affecting the different components of VILI pathophysiology (Nieman et al., 2017; Tonetti et al., 2017; Pinto et al., 2020). DP_L_ is the pressure to which the lung parenchyma is cyclically exposed during tidal breathing and represents the stress applied to the lung, not considering the pressure needed to overcome the chest-wall resistance. Since pleural pressure is not easily accessible to the bedside, Peso has been classically used to indirectly calculate transpulmonary pressure (Talmor et al., 2008; Beitler et al., 2019; Scaramuzzo et al., 2020a). However, Peso monitoring is invasive, can be technically challenging or not feasible in some patients, and requires precise calibration and interpretation. By exploring the intra-patient correlation of EIT-derived parameters and DP_L_, we found that a change in DP_L_ correlates negatively with a change of ventilation distribution in ROI1 and ROI2 while positively with a change in ROI3 and ROI4. This means that, when changing PEEP in patients with ARDS, an increase in relative ventilation in the dependent lung is related to a rise in DP_L_. This can be explained by the increased lung deformation which is related to a dorsal shift of ventilation but raises some questions on PEEP titration aiming to maximize dorsal ventilation (Pelosi et al., 2018). The association was even stronger when analyzing regional compliance, especially in the ROI2, which corresponds to the central-ventral ROI. Surprisingly, no significant correlation was found between change DP_L_ and regional compliance in ROI4, meaning that the esophageal-derived DP_L_ is less informative than thought on the regional characteristics of the dorsal part of the lung, despite the dorsal lung has been classically associated with the concept of PEEP titration guided by transpulmonary pressure. The central lung region corresponds to the position where the esophagus and, therefore, the esophageal catheter are supposed to be and explains why the stronger correlations have been found in the central lung ROIs.

We found that by integrating EIT and airway opening pressure information, DP_L_ can be predictable with good reliability and low bias. Specifically, esophageal derived DP_L_ was derived from IBW, Ers, and the relative ventilation in ROI1, ROI2, and ROI3. This is not the first attempt to evaluate non-invasively DP_L_ and EL. Lundin et al. (2015) recently introduced a method to estimate transpulmonary pressure from changes in end-expiratory lung volume (ΔEELV) following a PEEP step. Despite this approach is intriguing, it does need to perform a PEEP titration including the need to reach low or even zero PEEP. This is not problematic in patients undergoing general anesthesia which is the context of method validation but is less feasible in patients with ARDS, where the removal of PEEP can cause clinically important effects. Recently, Yoshida et al. (2018) demonstrated how transpulmonary pressure calculated using the subtractive method from Peso reflects the local behavior of the central to dependent lung. We hereby confirm this finding. Is has to be tested if this approach could be used to evaluate directly measured non-dependent lung DP, being at the moment still not possible to estimate local end-expiratory transpulmonary pressure and, therefore, the corresponding DP_L_ of this area.

We evaluated if also the inspiratory transpulmonary pressure, calculated using the elastance-derived method (Grasso et al., 2012) could be predicted by EIT. This parameter, indeed, has been demonstrated to be highly indicative of the transpulmonary pressure directly measured in the non-dependent lung (Yoshida et al., 2018). We found that EIT could predict its value with high accuracy, just by deriving lung and chest wall elastances by P_L, EIT_. This simple calculation, if implemented on available bedside EIT machines, would allow having continuously and non-invasively a good predictor of non-dependent transpulmonary pressure and, therefore, of the risk of barotrauma in this part of the lung.

We demonstrated that by using EIT data, it is possible to quantify DP_L_ and EL, as commonly calculated by the esophageal balloon. The immediate advantage of this is the possibility of measuring DP_L_ continuously and in patients in which the esophageal catheter positioning is technically challenging or the signal is not reliable. Moreover, we confirmed that P_L_ reflects the behavior of the central regions of the lung. Future studies need to evaluate if EIT can be used to calculate transpulmonary pressure in the other lung regions, allowing therefore to have at the bedside, regional transpulmonary pressure data. This information is currently not derivable by any non-invasive monitoring tool and could be precious in assessing regional early indicators of VILI.

This study has some limitations. First, it is derived from a limited number of observations and a small number of patients enrolled in two centers. Second, we used only one EIT machine to retrieve the percentage of relative ventilation in the ROI which is implemented with lung contouring based on the anthropometric characteristics of the patient. If this approach and the parameters derived in the regression equation can be applied also to the other EIT devices has to be confirmed (Lionheart, 2004). Third, in the protocol, we explored the correlation between DP_L_ measured using the esophageal balloon and EIT, by using a database of repeated measures at different levels of PEEP. Since regional DP can be modified by TV, future studies need to evaluate the impact of this parameter on regional transpulmonary pressure and the agreement between the two techniques. Finally, the population was characterized by patients affected mainly by ARDS associated with pneumonia or sepsis. None of the patients had a highly asymmetrical ARDS. The replicability of the findings must be, therefore, explored in this specific form of ARDS, due to the highly variable local forces, especially for their influence on the esophageal balloon signal (as shown in Supplementary Figure S3). No patient with COVID-19 was enrolled in this study and, therefore, also the applicability of this technique to patients with COVID-19 has to be tested. In conclusion, DP_L_ correlated with EIT-derived regional parameters, especially in the central lung. DP_L_, EL, and inspiratory transpulmonary lung pressures can be non-invasively estimated by integrating EIT-derived and airway opening pressure-derived data.

## Data Availability Statement

The data that support the findings of this study are available from the corresponding author upon reasonable request.

## Ethics Statement

The studies involving human participants were reviewed and approved by Sant' Anna Hospital, Ferrara, Italy (Protocol no. 171098) and Milan (protocol no. 625_2018). The patients/participants provided their written informed consent to participate in this study.

## Author Contributions

GS, SS, CV, and TM conceived of and coordinated the study. AW participated in its design and helped to draft and review the manuscript. IO, EM, and LG contributed to the interpretation of data and were involved in revising the manuscript. AW, SB, FM, and ES contributed to analysis and interpretation of data and were involved in revising the manuscript. AW contributed technical help during data analysis and revision of the final manuscript. GS and SS performed the statistical analysis and helped to carry out the data analysis. All authors meet all authorship requirements of the International Committee of Medical Journal Editors. All authors read and approved the final manuscript.

## Conflict of Interest

The authors declare that the research was conducted in the absence of any commercial or financial relationships that could be construed as a potential conflict of interest.
