# Supplementary material for: Calculation of Transpulmonary Pressure From Regional Ventilation Displayed by Electrical Impedance Tomography in Acute Respiratory Distress Syndrome
Source: Front Physiol. 2021 Jul 19;12:693736. doi: 10.3389/fphys.2021.693736 (PMC8327175; doi:10.3389/fphys.2021.693736)
Supplement: Supplementary file 1 [file Data_Sheet_1.pdf]

Calculation of transpulmonary pressure from regional ventilation displayed by Electrical Impedance Tomography in ARDS

Gaetano Scaramuzzo[1], Savino Spadaro[1], Elena Spinelli[2], Andreas D Waldmann[3], Stephan H. Bohm[3], Irene Ottaviani[1], Federica Montanaro[1], Lorenzo Gamberini[3], Elisabetta Marangoni[1], Tommaso Mauri[2,5], Carlo Alberto Volta[1]

SUPPLEMENTAL MATERIAL

Table S1:Regression analysis results

	Estimate	std.error	statistic	p.value
(Intercept)	16.63804	5.116479	3.251853	0.001997
RS elastance	0.230941	0.015528	14.87259	1.47E-20
%Roi1 (%)	-0.21449	0.07169	-2.99197	0.004201
%Roi2 (%)	-0.15974	0.035027	-4.56061	3.05E-05
%Roi3 (%)	-0.32996	0.079117	-4.17054	0.000113
IBW (kg)	0.074683	0.03122	2.392132	0.020329
R squared	*Adjusted R.squared*	*Statistic*	*p-value*	*deviance*
0.842983	0.82817	56.90841	4.24E-20	85.24723

Dependent variable: transpulmonary driving pressure (cmH_2O). Regressors: IBW, respiratory system elastance (RS elastance), % of ventilation in ROI1 (%ROI1), ROI2 (%ROI2), ROI3 (%ROI3) and ROI4 (%ROI4).

Figure S2: EIT-derived versus esophageal-derived transpulmonary pressure according to the different PEEP titration techniques.

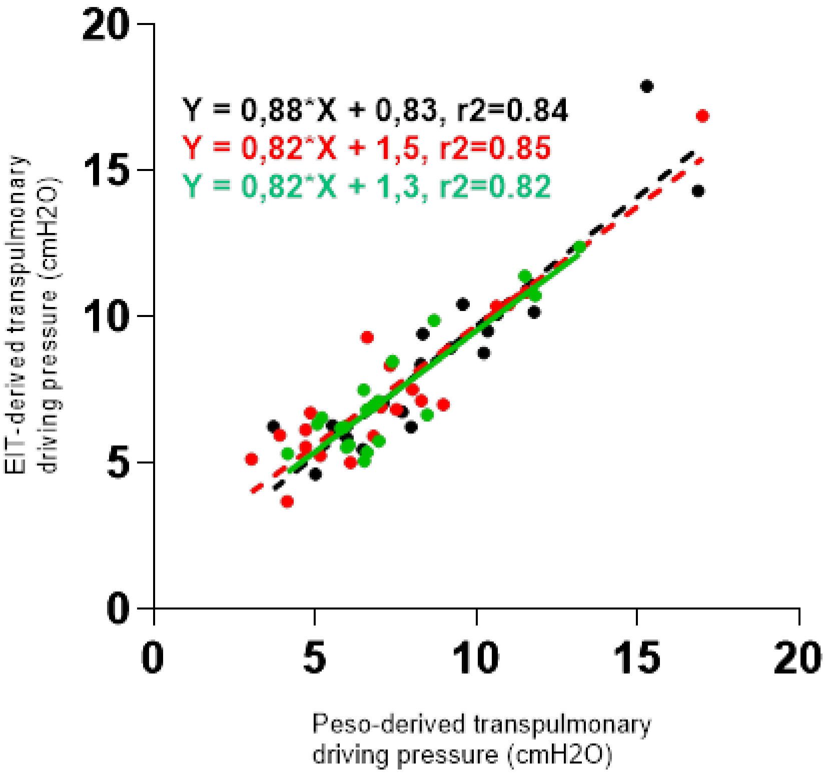

Regression of EIT derived transpulmonary pressure and Esophageal derived transpulmonary pressure in the three different PEEP titration settings (green= baseline; red=EIT; black=transpulmonary pressure).

Figure S3: regression analysis between EIT and Peso derived transpulmonary pressure in patients affected by 2 or 3-4 quadrants infiltration at the chest x-ray.

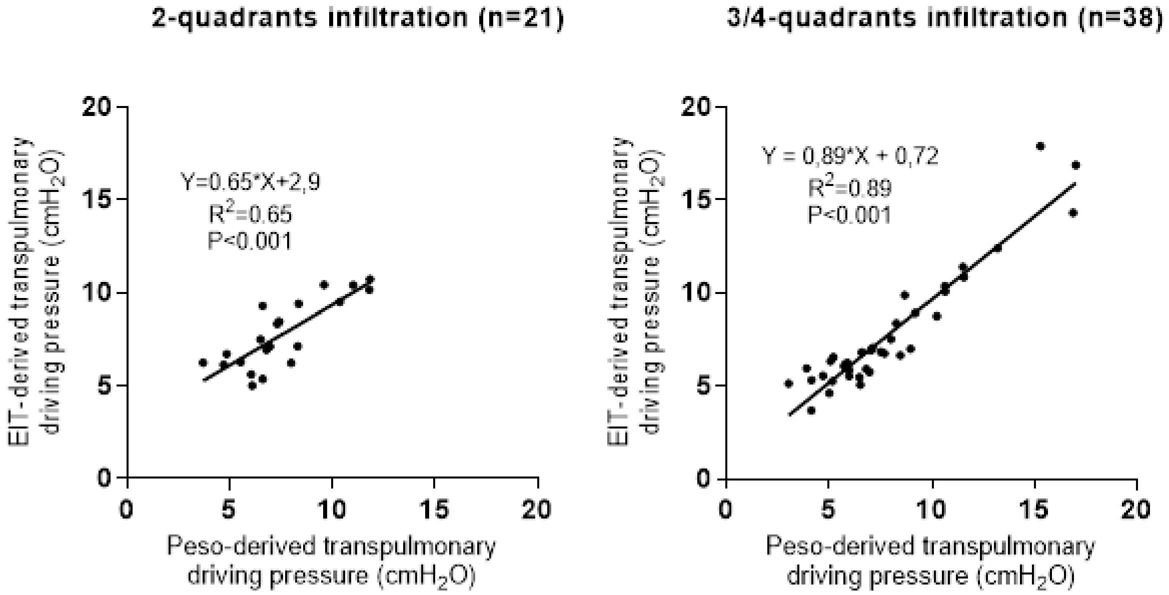

Left figure: regression between EIT and Peso derived transpulmonary pressure in patients affected by 2 quadrants infiltration at the x-ray. Right figure: regression between EIT and Peso derived transpulmonary driving pressure in patients affected by 3-4 quadrants infiltration at the x-ray. The regression is stronger when the infiltration is 3+. N= number of measures. EIT= electrical impedance tomography. Peso= Esophageal pressure.
